# Application of Artificial Intelligence in the Establishment of an Association Model between Metabolic Syndrome, TCM Constitution, and the Guidance of Medicated Diet Care

**DOI:** 10.1155/2021/5530717

**Published:** 2021-04-30

**Authors:** Pei- Li Chien, Chi-Feng Liu, Hui-Ting Huang, Hei-Jen Jou, Shih-Ming Chen, Tzuu-Guang Young, Yi-Feng Wang, Pei-Hung Liao

**Affiliations:** ^1^Public Affairs Office, Taiwan Adventist Hospital, No. 424, Sec. 2, Bade Road., Songshan District, Taipei City 10556, Taiwan; ^2^School of Nursing, National Taipei University of Nursing and Health Sciences, No. 365, Ming-te Road, Peitou District, Taipei City 112, Taiwan; ^3^Department of Gastroenterology, Taiwan Adventist Hospital, No. 424, Sec. 2, Bade Road., Songshan District, Taipei City 10556, Taiwan; ^4^Department of Obstetrics and Gynecology, Taiwan Adventist Hospital, No. 424, Sec. 2, Bade Road., Songshan District, Taipei City 10556, Taiwan; ^5^Department of Infectious Disease, Taiwan Adventist Hospital, No. 424, Sec. 2, Bade Road., Songshan District, Taipei City 10556, Taiwan; ^6^Health Clinic, Taiwan Adventist Hospital, No. 424, Sec. 2, Bade Road., Songshan District, Taipei City 10556, Taiwan

## Abstract

**Background:**

This study conducted exploratory research using artificial intelligence methods. The main purpose of this study is to establish an association model between metabolic syndrome and the TCM (traditional Chinese medicine) constitution using the characteristics of individual physical examination data and to provide guidance for medicated diet care.

**Methods:**

Basic demographic and laboratory data were collected from a regional hospital health examination database in northern Taiwan, and artificial intelligence algorithms, such as logistic regression, Bayesian network, and decision tree, were used to analyze and construct the association model between metabolic syndrome and the TCM constitution. *Findings*. It was found that the phlegm-dampness constitution (90.6%) accounts for the majority of TCM constitution classifications with a high risk of metabolic syndrome, and high cholesterol, blood glucose, and waist circumference were statistically significantly correlated with the phlegm-dampness constitution. This study also found that the age of patients with metabolic syndrome has been advanced, and shift work is one of the risk indicators. Therefore, based on the association model between metabolic syndrome and TCM constitution, in the future, metabolic syndrome can be predicted through the syndrome differentiation of the TCM constitution, and relevant medicated diet care schemes can be recommended for improvement.

**Conclusion:**

In order to increase the public's knowledge and methods for mitigating metabolic syndrome, in the future, nursing staff can provide nonprescription medicated diet-related nursing guidance information via the prediction and assessment of the TCM constitution.

## 1. Introduction

In 2018, the Statistics Department of the Ministry of Health and Welfare of Taiwan released the top 10 causes of death: cancer and metabolic syndrome are the major threats to the health of both men and women, and they also impose a great burden on health care expenditures and families [1, 2]. Metabolic syndrome criteria are (1) abdominal obesity: waist circumference ≥90 cm (35 inches) for men and ≥80 cm (31 inches) for women; (2) high blood pressure: systolic blood pressure ≥130 mmHg or diastolic blood pressure ≥85 mmHg; (3) high fasting blood glucose: fasting blood glucose value ≥100 mg/dl; (4) high triglyceride: ≥150 mg/dl; and (5) high-density lipoprotein cholesterol being low: male <40 mg/dl, and female <50 mg/dl. If more than three (including) of the above five factors are met, it can be determined as metabolic syndrome [3]. According to the International Diabetes Federation, about a quarter of people worldwide have metabolic syndrome, and the World Health Organization (WHO) predicts that diabetes will become the seventh leading cause of death by 2030 [4]. In 2017, the Word Federation of Obesity predicted that the number of obese population will reach 2.7 billion by 2025 [1, 5]. Many studies have pointed out that metabolic syndrome and bad lifestyle account for 50%. The high-risk groups for metabolic syndrome include those with a family history of hypertension, diabetes, and dyslipidemia, which accounts for about 20%, the incidence of metabolic syndrome of whom is higher than that of the general population, and people with a dietary preference for low fiber, high sugar, high fat, and excessive alcohol consumption, as well as stress, which results in endocrine disorders and increasing blood glucose [6]. Related literature points out that metabolic syndrome is one of the factors causing chronic diseases, such as cerebrovascular disease, heart disease, diabetes, hypertension [7]. For the proportion of patients with metabolic syndrome complicated by acute myocardial infarction, the proportion and mortality rates of major cardiovascular events were higher [5]. In order to achieve the goal of weight loss and thin waist circumference [8], Taiwan officially launched the metabolic syndrome prevention and control strategy in 2018, which aims at primary prevention to help people reduce the risk factors of metabolic syndrome and change their lifestyle, such as regular daily life habits, more intake of high-fiber, less oily and more healthy diet, and reducing animal fat and refined sugar intake.

The Chinese demand for preventive medicine and medical care is increasing. According to the World Health Organization's Global Strategy for Traditional and Alternative Medicine, 80 percent of the world's population still chooses traditional Chinese medicine as the primary medical care to some extent [9]. The US National Institutes of Health (NIH) classified complementary and alternative medicine (CAM) into five categories: (1) alternative medical system: traditional Chinese medicine, acupuncture, qigong, and so forth.; (2) psychosomatic-physical intervention therapy: meditation, hypnosis, music, and art therapy; (3) basic biological therapy: including traditional Chinese medicine diet therapy; (4) massage therapy; (5) energy therapy: diagnosing and treating diseases through energy fields [10]. The Taiwanese generally have the traditional concept of “treating disease or strengthening health without disease”; thus, CAM is becoming more and more popular when choosing auxiliary alternative medical treatment [11].

## 2. TCM Constitution and Medicated Diet

Western medicine divides the causes of disease into external and internal factors. Traditional Chinese medicine (TCM) believes that internal factors are closely related to the strength of the TCM constitution. The theory of the TCM constitution puts forward that the rise and fall of human vital qi can determine the strength of human disease-resistant ability; human vital qi is closely related to the individual constitution, which is the determinant factor of individual susceptibility to disease; and those with strong, vigorous, and healthy qi are not easily infected, even if there is epidemic disease [12]. For patients with the disease, the tendency to change their lifestyle due to the disease is also closely related to the strength of their constitution [13, 14]. Traditional Chinese medicine emphasizes physical conditioning to prevent the occurrence of diseases and promote rehabilitation; thus, the constitution plays an important role in the prevention and treatment of diseases in traditional Chinese medicine. While personal constitution is closely related to sickness tendency, constitution can be changed under certain conditions [15]. Personal constitution is affected by genetic factors, social environment, natural environment, work status, diseases, drugs, and diet [16]. The constitution theory was established from Neijing, Article 47 of Viscera, Volume 7 of Miraculous Pivot of Viscera in Huangdi Neijing, which is an ancient book that states the five viscera containing spirit, vigor, and soul, while the six hollow organs digest food and excrete body fluids [17]. That is, the essence, qi, blood, body fluids, etc. that are required for human life are stored in the five viscera and six hollow organs to operate and breathe [17]. Constitution refers to the stable form of the human body, which is inherited by nature and influenced by various acquired factors, and adapts to the natural and social environment. In traditional Chinese medicine, the constitution is divided into peace, qi deficiency, yang deficiency, yin deficiency, blood deficiency, damp-heat, qi stagnation, blood stasis, special constitution, etc. [17]. The constitution theory is the theoretical knowledge of studying different physiological characteristics and analyzing these characteristics according to disease occurrence, life extension, and reaction state. However, the above life processes have their own particularities for different individuals, such as health preservation and disease prevention [13, 17]. The connotations of the constitution include body, physique, characteristics, and quality, as well as physiological, pathological, and psychological characteristics. Physiological and pathological characteristics refer to the particularities and differences of individual external morphology and internal physiology, the metabolic function in the growth and development process, and the prognosis and spread of disease after the onset of disease. Nowadays, physiological and pathological trends are dominant [18].

In traditional medicine, the principle of prolonging life is to drink to nourish yang, eat to nourish yin, eat less food, and not cause deficiency. In the same way, we should have more meals a day, but less food at each meal, avoid refined and fatty foods, and follow the rule of low-salt and high-fiber diets. The most common metabolic syndrome is cardiovascular stroke, and its pathological basis is arteriosclerosis, which is related to hyperlipidemia and arteriosclerosis, and its prevention and treatment have received worldwide medical attention [19, 20]. In the antiaging diet, foods that can reduce blood lipid are as follows: agaric, Chinese dates, longan, wolfberry, angelica [19, 21], mushroom, beans, onion, ginger, garlic, radish, water cabbage, cauliflower, eggplant, kelp, hawthorn, milk, oats, apple, sesame, etc. Fish can strengthen the spleen and replenish qi, and protein is rich in methionine, lipoprotein, and unsaturated fatty acid, which can promote excretion, absorption, and lower blood pressure [22]. Green tea and ginkgo biloba are natural antioxidants that have been widely studied to reduce blood lipid and prevent platelet aggregation [23]. Poria cocos and caulis spatholepis have antitumor effects [24]. According to the theory of traditional Chinese medicine, “the heart stores the spirit,” “the kidney governs the bone to produce marrow,” and “the brain is the sea of marrow and the house of spirit.” According to modern pharmacology research, the deficiency of qi in the heart and kidneys, as well as the loss of the main bone marrow, should be treated with nootropic drugs, such as ripe Rehmannia, *ginseng*, *Codonopsis codonopsis*, *Acanthopanax senticosus*, jujube, yam, astragalus, dodder, *Eucommia ulmoides*, and *Uncaria* [21], which can expand the volume of cerebral circulation, enhance brain memory, and restore the body from fatigue [25].

## 3. Artificial Intelligence

Artificial intelligence (AI) refers to the intelligence shown by machines after learning from humans. Human-like intelligence technology, which builds knowledge and experience on computers and realized through computer programs, was formally named by computer scientist John McCarthy in 1956 [26]. Artificial intelligence attempts to mimic or enhance human intelligence and enables the development of highly complex communications, such as easy-to-use human-computer interfaces, customized text message alerts for individual patients, sleep, exercise, and glucose monitors, and avatars acting as health coaches [27]. In 2010, Kreps and Neuhauser mentioned that, as health information technology grows, artificial intelligence designs feature interactive, easy-to-use e-health applications that convey the correct information needed to guide health care and promote healthy outcomes to the public [28]. Scholars have also proposed that artificial intelligence could be used to build big data analysis and cloud platform care systems, introduce intelligent medical-related information technology equipment, and create decision-making care information systems with integrated software systems [29]. In the future, the Internet of Things will be used to develop medical sharing platforms, where the portable devices on the patient side will be developed to automatically record personal physiological health data; the eHealth database on the medical institution side will be automatically retrieved to facilitate a larger and more meaningful range of information; and then, a medical decision-making system knowledge base can be established [30, 31]. Machine learning is the product of artificial intelligence crowd thinking, and its algorithms include decision tree learning, inductive logic programming, clustering, reinforcement learning, and Bayesian network [32].

## 4. Methodology

### 4.1. Research Design and Process

This study was designed as exploratory research. More than 8,316 medical records of health examination cases were extracted from HIS (Health Information System) in a regional teaching hospital in northern Taiwan according to CRISP (Cross-Industry Standard Process for Data Mining) procedures. First, the incidences of risk factors affecting the metabolic syndrome were discussed, as based on relevant literature. After comparison and verification of literature regarding traditional Chinese medicine and Western medicine, the main judgment factors were, respectively, selected from the case data sets and integrated into this study as variables to construct the correlation between TCM syndromes and metabolic syndrome, and the nursing guidance of integrated TCM and western medicine was proposed according to the principles of Chinese medicinal diet recommendations.

### 4.2. Research Subjects and Data Set

This study collected the medical records of health examinations from a regional hospital in northern Taiwan from April 1, 2016, to March 31, 2018. The subjects of this study were between 20 and 80 years old. The collected columns, including the original continuous values and the columns classified as normal and abnormal values, are as follows: (1) persons with abnormal body mass index (BMI) ≧24 kg/m^2^, (2) men with abnormal waist circumference ≧90 cm, women with abnormal waist circumference ≧80 cm, (3) persons with abnormal blood pressure (systolic blood pressure ≧130 mmHg, diastolic blood pressure ≧85 mmHg), (4) persons with abnormal blood glucose before meal ≧126 mg/dl, (5) persons with abnormal total cholesterol ≧200 mg/d, (6) persons with abnormal low-density lipoprotein (LDL-C) ≧130 mg/dl, (7) persons with abnormal high-density lipoprotein (HDL-C) ≦40 mg/dl, and (8) persons with abnormal triglyceride ≧160 mg/dl. The data includes basic information (number, age, gender, arteriosclerosis test, blood pressure, and liver function), symptoms (about 15 attributes), and examination items (13 attributes). All column data was analyzed by IBM SPSS for Windows 25.0, and then, the classification prediction model was established with IBM SPSS Modeler 18.0 through machine learning.

### 4.3. Data Analysis

The machine learning classification and prediction techniques used in this study are mainly binary logistic regression, the Bayesian network conditional probability algorithm, and the decision tree analysis method. Binary logistic regression is a kind of classification algorithm [33], which is widely used by generalized regression algorithms for classification problems. A linear classifier called regression is a variation of linear regression, the solution of which enables the model to obtain the value of $*W*$, as well as the parameter with the highest degree of data fit. The expression for linear regression is used as the input for logistic regression and is an iterative algorithm like linear regression, where the cost function is continuously optimized by iteration and weight $*W*$ is updated by introducing the link function. The linear regression equation $*Z* (*x*)$ is converted to $*g*(*z*)$, so that the value of $*g*(*z*)$ is distributed between 0 and 1, and the classification model is obtained. When $*g*(*z*)$ is close to 0, the label of the sample is 0; when $*g*(*z*)$ is close to 1, the label of the sample is 1. This link function is the Sigmoid function, which is only applicable in the binary classification scenario [34].

The Bayesian network, also known as a belief network or directed acyclic graphical model, is a probability graph model financed by a directed acyclic graph. The properties of a group of random variables and their *n* groups of conditional probability distributions (CPDs) are known from the directed acyclic graphs (DAGs) (equation (1)) [35]. A Bayesian network can be used to represent the probability relationship between a disease and its related symptoms. Given a known symptom, the Bayesian network can be used to calculate the probability of various possible diseases [36]. Generally speaking, nodes in the directed acyclic graphs of a Bayesian network represent random variables, which can be observed variables, potential variables, unknown parameters, and so forth. The arrows connecting two nodes indicate that the two random variables are causally or unconditionally independent; if there are no arrows connecting two nodes, the random variables are said to be conditionally independent; if two nodes are connected by a single arrow, it means that one of the nodes is a “parent” and the other is a “descendant or child,” and these two nodes will produce a conditional probability value [37]:

(1) $$\begin{array}{c} f_{t}\left\{X_{1},X_{2},\ldots ,X_{n}\right\}. \end{array}$$

The tree structure of the decision tree is that the intermediate node of the tree is the test condition, the branch of the tree is the result of the conditional testing, and the leaf node of the tree is the result of classification. Each node of a decision tree is a judgment formula to determine whether the input data conforms to this path according to a variable. Therefore, each node can divide the input data into several categories, while the leaf nodes of the tree represent the classification result after classification [35, 36]. The CHAID (Chi-Square Automatic Interaction Detector) algorithm uses the Chi-Square test to predict whether two variables need to be combined; if it can generate the predictive variable with the largest category difference, it will become the separated variable of the node. Calculate the *P* value of the category in the node to determine whether or not the decision tree continues to grow, and CHAID stops the expansion of fulcrum sprawl before excessive application [26].

## 5. Results

### 5.1. Demographic and Laboratory Data Analysis

The basic data of the 8,316 subjects in this study was analyzed as follows: they were aged 23–82 years, with an average age of 45.44, an average height of 168.74 cm, an average weight of 69.76 kg, and an average BMI of 24.22 kg/m^2^, and their occupations included commerce, service industry, industry, manufacturing industry, science, and technology industry. The majority were males (70.2%), and the average waist circumference was 81.21 cm (81.21 ± 10.81) and 85.26 cm (85.26 ± 8.92) for men and 74 cm (74 ± 4.54) for women. In terms of blood pressure, the mean systolic blood pressure was 126.11 mmHg (126.11 ± 16.69), and the mean diastolic blood pressure was 77.92 mmHg (77.92 ± 11.08). The cholesterol was 200.13 mg/dL (SD = 41.73). The blood biochemical values associated with laboratory tests were 6.28 mg/dL for white blood cells (WBC), 14.96 mg/dL for hemoglobin (Hb), 46.91 mg/dL for high density cholesterol (HDL), 124.18 mg/dL for low-density cholesterol (LDL), 144.79 mg/dL for triglyceride (TrG), 0.92 mg/dL for creatinine (Cr), 3.76 mg/dL for red blood cells (RBC), 86.65 mg/dL for platelets (PLT), 7.75 mg/dL for blood urea nitrogen (BUN), 95.09 mg/dL for blood glucose before meal (Glu AC), 3.69 mg/dL for hemoglobin glycosylated (HbA1C), 61.88 mg/dL for hepatitis B virus, 2.50 mg/dL for hepatitis C virus antibody virus, and 2.99 mg/dL for hepatitis A virus. The physical examinations and assessment systems were HEENT, respiration system, cardiovascular system, nervous system, skin system, etc., and more than 80% were normal. The frequency of metabolic syndrome components occurring in the health examination database was further analyzed, and the most common factor was excessive waist circumference, followed by fasting blood glucose, low HDL cholesterol, and hypertension, respectively (as shown in Table 1).

In this study, 3 professional TCM physicians judged the TCM constitutions corresponding to the relevant parameters and indices and found that the phlegm-dampness and damp-heat constitutions ranked highest in the TCM constitutions of the groups with a high risk of metabolic syndrome, followed by yin deficiency, qi deficiency, and qi stagnation constitutions, respectively. The results of Chi-Square testing show that cholesterol, waist circumference, and blood glucose were statistically significantly correlated with phlegm-dampness, yin deficiency, yang deficiency, damp-heat, and other constitution issues (as shown in Table 2).

### 5.2. Association Model between Metabolic Syndrome and TCM Constitution Prediction

Among the 8,316 data collected, it was found that blood glucose before meal, total cholesterol, waist circumference, age, hypertension, and shift work were more important than other test factors. Therefore, this study used these 6 high-risk factors to analyze the correlation and prediction of 4 major TCM constitutions.

*Logistic Regression*. According to logistic regression analysis, the overall prediction rates of the 6 high-risk factors for TCM constitution were 88.9% for phlegm-dampness, 88.8% for yin deficiency, 65.4% for yang deficiency, and 91.3% for damp-heat. According to the highest prediction rate of damp-heat constitution, the best correlation factors were hypertension, abnormal waist circumference, hyperglycemia, and shift work, which were statistically significant (*P* < 0.05). According to the relative risk odds ratio, people without hypertension are 0.876 times more likely to belong to the damp-heat constitution than those with hypertension. That is, people without shift work are 0.702 times more likely to belong to the damp-heat constitution than those with shift work (as shown in Table 3).

**Figure 1:**
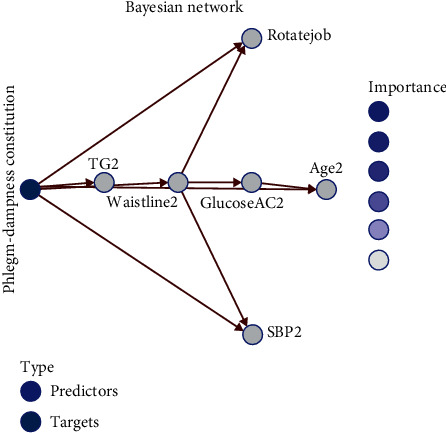
Prediction using the conditional probability analysis for each constitution as shown by the Bayesian network.

*Bayesian Network*. According to Bayesian network analysis, the overall probability of the six high-risk factors, as predicted by the conditional probability analysis for each constitution, reached more than 80%, and only the yang-deficiency constitution failed to reach 70%. The prediction rate of the phlegm-dampness constitution was 89.1%, and the main influencing factors were excessive waist circumference and hyperglycemia. Based on conditional probability analysis, the prediction rate of the 6 high-risk factors for the phlegm-dampness constitution was 65.1%, and the main influencing factors were high cholesterol and hyperglycemia (Figure 1).

**Figure 2:**
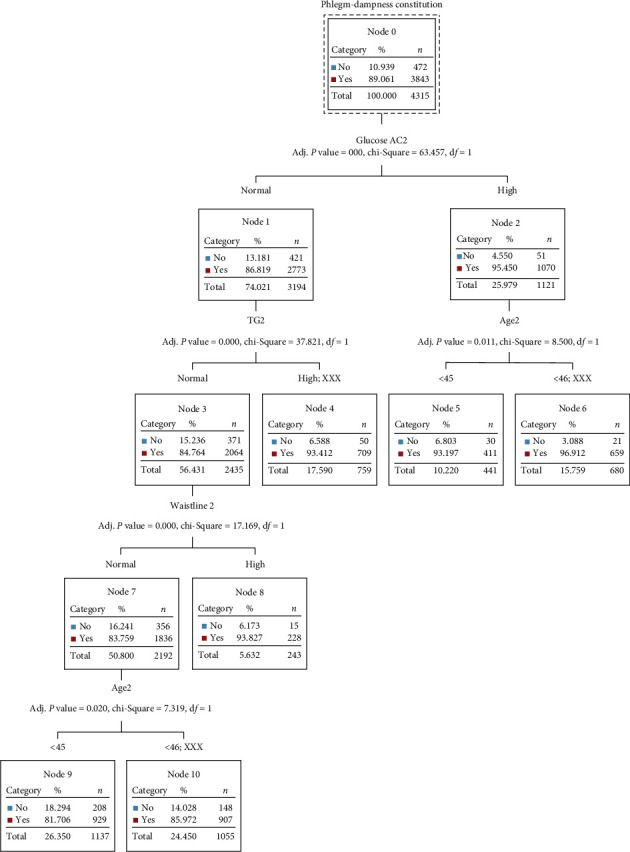
Decision tree.

CHAID (Chi-Square Automatic Interaction Detector). According to decision tree analysis, the prediction rate of the 6 high-risk factors for the phlegm-dampness constitution was the highest at 89.1%, and the main influencing factors were hyperglycemia and those aged above 45 years old. The prediction rate of the 6 high-risk factors for yang deficiency constitution was the lowest at 77.1%, and the main influencing factors were excessive waist circumference, hyperglycemia, and age above 45 years old (Figure 2).

## 6. Discussion

### 6.1. Correlation between Occupation, Metabolic Syndrome, and TCM Constitution

According to the study of metabolic syndrome and occupational categories, as conducted in 2009 by Executive Yuan labor safety researchers, the prevalence rate of metabolic syndrome among male workers in Taiwan was 19.4%, while that among female workers was 14.8%, with the highest rates among commerce, manufacturing, and construction industries. The related risk factors were hyperglycemia, high cholesterol, sleep quality, and shift work, which are different from the results of this study. This study shows that the technology industry and shift workers suffer the most metabolic syndrome, and the main risk factors were hyperglycemia, high cholesterol, and excessive waist circumference, which may be different from changes in social patterns [38]. The results of this study are similar to the research results of other scholars. It was found that women with a sedentary work style had significantly higher hypertension, fasting blood glucose, and total cholesterol than women with a nonsedentary work style and that physical activity at work and sitting, standing, and walking time at work were significantly correlated with BMI, static blood pressure, and total cholesterol [39]. In addition, other scholars discussed the comparison between work and cardiovascular disease and showed that the risk of cardiovascular disease was 17.5 times higher in the blue-collar class than in the professional or managerial class [40]. According to the theory of the TCM constitution and clinical experience, many factors are directly or indirectly related to the constitution. In order to explore the correlation analysis between cholesterol and the TCM constitution, this study found statistically significant differences in risk factors related to the phlegm-dampness constitution (*P* < 0.05) and metabolic syndrome [41]. According to traditional Chinese medicine diagnosis literature, a yin deficiency constitution and phlegm-dampness constitution are prone to hypertension, which is similar to the results of this study [17, 25]. According to the 2015 Human Nutrition and Health Survey, the prevalence rate of metabolic syndrome was 36.6% for men and 57.5% for women. The prevalence rate of metabolic syndrome was positively correlated with age; after the age of 45, the risk of metabolic syndrome increased sharply for women, and the prevalence rate peaked for both men and women in the age range of 51–60 years [42]. The results of this study show that the average age onset of metabolic syndrome in men was 46.24 years old, and the incidence rate in women was 43.54 years old. Compared with other literature, there is a risk of early onset of metabolic syndrome.

### 6.2. TCM Constitution and Medicated Diet

This study shows that the phlegm-dampness constitution and damp-heat constitution are the most common TCM constitutions of metabolic syndrome. Hyperglycemia, high cholesterol, excessive waist circumference, and shift work are the most important factors affecting metabolic syndrome. The recommendation guidelines for the TCM constitution and medicated diet mention that the cases of phlegm-dampness constitution are mostly manifested as having a preference for greasy food, slow reaction, and mental tiredness, and it is suggested to take kelp coix seed soup, almond porridge, or loquat leaf balloon flower tea. After clinical evidence, the cases subjectively indicated a continuous improvement in symptoms [43].

### 6.3. Recommended Medicated Diet for the Nursing Model of Integrated TCM and Western Medicine

Although research on the subhealth state of metabolic syndrome has made progress, there is no specific assessment scale for the metabolic syndrome and TCM constitution. One of the unique functions of nursing staff [44] is health assessment and guidance. If nursing staff learn knowledge about TCM constitution assessment and medicated diet, then the TCM constitution prediction results may be developed in the future and combined with the nursing guidance model recommended by medicated diet. The empirical nursing verification for this nursing model of integrated TCM and western medicine can then be carried out to provide new ideas for the nursing model of metabolic syndrome.

## 7. Conclusion

This study found that the age of patients with metabolic syndrome has been gradually getting younger, which is worthy of early lifestyle change. In addition, it was found that shift work is a risk indicator, which should be paid more attention to in the future. In terms of a TCM constitution, the metabolic syndrome constitution is dominated by the phlegm-dampness constitution and damp-heat constitution. Future research can try to identify more predictive models for TCM constitution, metabolic syndrome, and other high-risk chronic diseases and provide related care, in order to improve physical conditioning through diet (traditional Chinese medicine diet), lifestyle, and rest. Nursing staff can also provide personalized TCM nursing information to achieve the concept of health promotion and early awareness medicine.

## Figures and Tables

**Table 1:** Demographic and laboratory data analysis (*N* = 8316).

Demographic characteristic	Mean (SD)	*N* (%)
Gender		
Male		3031 (70.1)
Female		1285 (29.7)
Age (years old)	45.44 (8.5)	
Occupation		
Commerce, service		713 (16.5)
Industrial manufacturing		1444 (33.5)
Technology industry		2158 (50)
Height (cm)	168.74 (8.0)	
Weight (kg)	69.76 (13.5)	
Waist circumference (cm)	81.2 (10.7)	
BMI (kg·m^2^)	24.22 (3.73)	
Systolic blood pressure (mm Hg)	122.6 (17.4)	
Diastolic blood pressure (mm Hg)	77.92 (11.5)	
Glucose (mg/dl)	95.17 (18.2)	
TC (mg/dl)	202.5 (35.7)	
HDL-C (mg/dl)	46.91 (13.6)	
LDL-C (mg/dl)	124.18 (30.9)	
TG (mg/dl)	144.79 (35.7)	
WBC (mg/dL)	6.28 (1.59)	
Hb (mg/dL)	14.96 (1.02)	
Creating (mg/dL)	0.92 (0.14)	
PLT (mg/dL)	86.65 (2.79)	
HbA1C (mg/dL)	3.69 (0.036)	
Hepatitis B virus (mg/dL)	61.88 (14.10)	
Hepatitis C virus (mg/dL)	2.50 (0.086)	
Hepatitis A (mg/dL)	2.99 (0.04)	

**Table 2:** Correlation between cholesterol and TCM constitution (Pearson chi-square test).

Variable	Value	d*f*	*P*
Phlegm-dampness constitution	355.907	1	0.000^*∗*^
Yin deficiency constitution	354.408	4	0.007^*∗*^
Yang deficiency constitution	458.620	1	0.000^*∗*^
Qi deficiency constitution	1044.382	1	0.273
Damp-heat constitution	374.334	1	0.000^*∗*^

^*∗*^ *P* < 0.05.

**Table 3:** Risk factors of metabolic syndrome predicting TCM constitution and risk assessment analysis.

Variable	Age		SBP		TG		Waistline		Glucose		Rota job		Total
	*P*	OR	*P*	OR	*P*	OR	*P*	OR	*P*	OR	*P*	OR	PR
Phlegm-dampness constitution	0.001^*∗*^	0.701	0.663	0.669	0.001^*∗*^	0.556	0.001^*∗*^	0.512	0.001^*∗*^	0.419	0.897	1.014	88.9
Yin deficiency constitution	0.001^*∗*^	0.706	0.632	0.944	0.001^*∗*^	0.566	0.001^*∗*^	0.510	0.001^*∗*^	0.417	0.874	1.018	88.8
Yang deficiency constitution	0.001^*∗*^	0.804	0.991	0.999	0.001^*∗*^	0.794	0.357	1.082	0.001^*∗*^	0.422	0.001^*∗*^	0.716	65.4
Qi deficiency constitution	0.330	0.657	0.001^*∗*^	0.876	0.336	0.496	0.001^*∗*^	0.418	0.001^*∗*^	0.412	0.001^*∗*^	0.702	91.3

^*∗*^ *P* < 0.05. PR: predicting rate. OR: odds ratio.

## Data Availability

The data that support the findings of this study are available from the corresponding author, upon reasonable request.

## References

[1] World Health Organization (2019). Health theme diabetes. https://www.medpartner.club›who-2019-health-threat-taiwan-aspect.

[2] World Health Organization (2019). Death cause statistics in 2019. https://money.udn.com/money/story/5648/3884903.

[3] Kassi E., Pervanidou P., Kaltsas G., Chrousos G. (2011). Metabolic syndrome: definitions and controversies. *BMC Medicine*.

[4] Rowley W. R., Bezold C., Arikan Y., Byrne E., Krohe S. (2017). Diabetes 2030: insights from yesterday, today, and future trends. *Population Health Management*.

[5] World Obesity Federation (2019). World obesity day. https://www.hpa.gov.tw/Pages/List.aspx?nodeid=3998.

[6] Zhu L., Spence C., Yang W. J., Ma G. X. (2020). The IDF definition is better suited for screening metabolic syndrome and estimating risks of diabetes in Asian American adults: evidence from NHANES 2011-2016. *Journal of Clinical Medicine*.

[7] Softic S., Cohen D. E., Kahn C. R. (2016). Role of dietary fructose and hepatic de novo lipogenesis in fatty liver disease. *Digestive Diseases and Sciences*.

[8] Teng T., Wen H. Y., Hsiao S. F. (2018). The effects of cardiopulmonary exercise intervention on acute ST elevation myocardial infarction with metabolic syndrome patient: a case report. *Formosan Journal of Physical Therapy*.

[9] Xin B., Mu S., Tan T., Yeung A., Gu D., Feng Q. (2020). Belief in and use of traditional Chinese medicine in Shanghai older adults: a cross-sectional study. *BMC Complementary Medicine and Therapies*.

[10] Fan K. W. (2005). National center for complementary and alternative medicine website. *Journal of the Medical Library Association*.

[11] National Cancer Institute at the National Institutes of Health (2020). Complementary and alternative medicine. https://www.cancer.gov/about-cancer/treatment/cam.

[12] Kohn L. (2011). Health maintenance in ancient China. *International Journal of Medical Sciences*.

[13] Lin J.-D., Lin J.-S., Chen L.-L., Chang C.-H., Huang Y.-C., Su Y.-C. (2012). BCQs: a body constitution questionnaire to assess stasis in traditional Chinese medicine. *European Journal of Integrative Medicine*.

[14] Wong Y. C. P. (2016). Need of integrated dietary therapy for persons with diabetes mellitus and “unhealthy” body constitution presentations. *Journal of Integrative Medicine*.

[15] Li L., Yao H., Wang J., Li Y., Wang Q. (2019). The role of Chinese medicine in health maintenance and disease prevention: application of constitution theory. *The American Journal of Chinese Medicine*.

[16] Liu J., Xu F., Mohammadtursun N., Lv Y., Tang Z., Dong J. (2017). The analysis of constitutions of traditional Chinese medicine in relation to cerebral infarction in a Chinese sample. *The Journal of Alternative and Complementary Medicine*.

[17] Li M., Mo S., Yubao Y., Tang Z., Dong J. (2017). A study of traditional Chinese medicine body constitution associated with overweight, obesity, and underweight. *Evidence-Based Complementary and Alternative Medicine*.

[18] Yao S., Zhang Z. Z., Yang X. S. (2012). Analysis of composite traditional Chinese medicine constitution: an investigation of 974 volunteers. *Journal of Chinese Integrative Medicine*.

[19] Lin J. C., Wu S. F., Hsu C. H., Lee M. S., Lin E. (2019). Analysis of differences between patients with metabolic syndrome and TCM syndromes. *Journal of Traditional Chinese Medical Sciences*.

[20] Montazerifar F., Bolouri A., Paghalea R. S., Mahani M. K., Karajibani M. (2016). Obesity, serum resistin and leptin levels linked to coronary artery disease. *Arquivos Brasileiros de Cardiologia*.

[21] Perng P. C., Chen W. L., Liao F. Y., Fang W. H., Chang Y. W. (2018). The association between metabolic syndrome, the associated biomarkers, and endothelial function. *Chinese Journal of Occupational Medicine*.

[22] Lees M. J., Carson B. P. (2020). The potential role of fish-derived protein hydrolysates on metabolic health, skeletal muscle mass and function in ageing. *Nutrients*.

[23] Sharma B. R., Kim D. W., Rhyu D. Y. (2017). Korean Chungtaejeon tea extract attenuates weight gain in C57BL/6J-Lep ob/ob mice and regulates adipogenesis and lipolysis in 3T3-L1 adipocytes. *Journal of Integrative Medicine*.

[24] Fan W. S., Mou M. L., Tsai L. H., Mei C. H. (2019). Application and research on raw materials of traditional Chinese medicine diet. *Asia Pacific Family Medicine*.

[25] Chang M. Y., Liu C. Y., Chu M. C. (2013). Conditions for the use of complementary and alternative medicine in Taiwan: a nationwide survey analysis for 2011. *Taiwan Journal of Public Health*.

[26] Lin M. F., Chen W. J., Chang X. L., Lai Y. W. (2014). Drug interactions build database of clinical classification. *Journal of Medical Quality*.

[27] Kao C. W., Chuang H. W., Chen T. Y. (2017). The utilization of health-related applications in chronic disease self-management. *Journal of Nursing*.

[28] Liu H. Y., Chen T. J. (2017). Current state and development of m-health app usage. *Clinical Medicine*.

[29] Ahuja A. S. (2019). The impact of artificial intelligence in medicine on the future role of the physician. *PeerJ*.

[30] Mehralian H. A., Moghaddasi J., Rafiei H. (2019). The prevalence of potentially beneficial and harmful drug-drug interactions in intensive care units. *Drug Metabolism and Personalized Therapy*.

[31] Min J. S., Bae S. K. (2017). Prediction of drug-drug interaction potential using physiologically based pharmacokinetic modeling. *Archives of Pharmacal Research*.

[32] Liao S. H., Wen C. H. (2019). *Data Mining: Artificial Intelligence and Machine Learning Development*.

[33] Simundic A.-M. (2013). Bias in research. *Biochemia Medica*.

[34] Burdack J., Horst F., Giesselbach S. (2020). A public dataset of overground walking kinetics in healthy adult individuals on different sessions within one day. *Mendeley Data*.

[35] Halilaj E., Rajagopal A., Fiterau M., Hicks J. L., Hastie T. J., Delp S. L. (2018). Machine learning in human movement biomechanics: best practices, common pitfalls, and new opportunities. *Journal of Biomechanics*.

[36] Phinyomark A., Petri G., Ibáñez-Marcelo E., Osis S. T., Ferber R. (2018). Analysis of big data in gait biomechanics: current trends and future directions. *Journal of Medical and Biological Engineering*.

[37] Burdack J., Horst F., Giesselbach S. (2020). Systematic comparison of the influence of different data preprocessing methods on the performance of gait classifications using machine learning. *Frontiers in Bioengineering and Biotechnology*.

[38] Li Y. S., Chi Y. C., Chang W. P., Wu C. L. (2010). The relationship between occupation and metabolic syndrome in Taiwan, Taipei City. *Medical Journal*.

[39] Al Mansari A., Obeid Y., Islam N. (2018). Goal study: clinical and non-clinical predictive factors for achieving glycemic control in people with type 2 diabetes in real clinical practice. *BMJ Open Diabetes Research & Care*.

[40] Majumdar A., Mitra A., Parthibane S., Revadi G. (2019). Young, obese, and underweight patients show up inadequately at scheduled appointments: findings from a record-based study on diabetic, hypertensive diabetic, and hypertensive patients attending a primary care clinic of Puducherry. *Journal of Family Medicine and Primary Care*.

[41] Hung R. L., Huang W. H., Chen H. L., Huang L. C. (2013). Association of weight gain in adulthood and metabolic syndrome. *Taiwan Journal of Family Medicine*.

[42] Huang L. W., Chen I. J., Hsu C. H. (2015). Influence of traditional Chinese medicine syndrome groups on quality of life in women with metabolic syndrome. *Journal of Traditional and Complementary Medicine*.

[43] Lee C.-H., Li T.-C., Tsai C.-I. (2015). Yang deficiency body constitution acts as a predictor of diabetic retinopathy in patients with type 2 diabetes: taichung diabetic body constitution study. *Evidence-Based Complementary and Alternative Medicine*.

[44] Choi K. S. (2020). Integrating artificial intelligence into healthcare research. *The Journal of Nursing*.

